# Midwall Fibrosis and Cardiac Mechanics: Rigid Body Rotation Is a Novel Marker of Disease Severity in Pediatric Primary Dilated Cardiomyopathy

**DOI:** 10.3389/fcvm.2021.810005

**Published:** 2022-02-17

**Authors:** Nadya Al-Wakeel-Marquard, Franziska Seidel, Jirko Kühnisch, Titus Kuehne, Felix Berger, Daniel R. Messroghli, Sabine Klaassen

**Affiliations:** ^1^Department of Congenital Heart Disease – Pediatric Cardiology, German Heart Center Berlin, Berlin, Germany; ^2^Institute of Computer-Assisted Cardiovascular Medicine, Charité – Universitätsmedizin Berlin, corporate member of Freie Universität Berlin and Humboldt-Universität zu Berlin, Berlin, Germany; ^3^DZHK (German Centre for Cardiovascular Research), Partner Site Berlin, Berlin, Germany; ^4^Experimental and Clinical Research Center, A Cooperation Between the Max-Delbrück-Center for Molecular Medicine in the Helmholtz Association and Charité – Universitätsmedizin Berlin, Berlin, Germany; ^5^Department of Pediatrics, Division of Cardiology, Charité – Universitätsmedizin Berlin, corporate member of Freie Universität Berlin and Humboldt-Universität zu Berlin, Berlin, Germany; ^6^Department of Internal Medicine – Cardiology, German Heart Center Berlin, Berlin, Germany; ^7^Department of Cardiology, Charité – Universitätsmedizin Berlin, corporate member of Freie Universität Berlin and Humboldt-Universität zu Berlin, Berlin, Germany

**Keywords:** midwall fibrosis, cardiac mechanics, rigid body rotation, cardiovascular magnetic resonance, dilated cardiomyopathy, pediatric

## Abstract

**Background:**

Midwall fibrosis (MWF) detected by late gadolinium enhancement (LGE) cardiovascular magnetic resonance (CMR) predicts adverse outcome in adults with dilated cardiomyopathy (DCM). Its relevance in children and adolescents is relatively unknown. Left ventricular (LV) strain, rotation and twist are important parameters of cardiac function; yet, their role in pediatric heart failure is understudied. This study aimed to evaluate MWF and cardiac mechanics in pediatric DCM.

**Methods:**

Patients ≤21 years with primary DCM were prospectively enrolled and underwent standardized CMR including LGE. All participants were categorized according to the presence or absence of MWF (MWF+ vs. MWF–). Cardiac mechanics were assessed using CMR feature tracking. Impaired LV twist with apex and base rotating in the same direction was termed rigid body rotation (RBR).

**Results:**

In total, 17 patients (median age 11.2 years) were included. MWF was present in seven patients (41%). Median N-terminal pro brain natriuretic peptide (NT-proBNP) was higher (5,959 vs. 242 pg/ml, *p* = 0.887) and LV ejection fraction (LVEF) lower (28 vs. 39%, *p* = 0.536) in MWF+ vs. MWF– patients, yet differences were not statistically significant. MWF+ patients had reduced global longitudinal (GLS), circumferential (GCS) and radial strain (GRS), again without statistical significance (*p* = 0.713, 0.492 and 1.000, respectively). A relationship between MWF and adverse outcome was not seen (*p* = 0.637). RBR was more common in MWF+ (67 vs. 50%), and was associated with the occurrence of adverse events (*p* = 0.041). Patients with RBR more frequently were in higher New York Heart Association classes (*p* = 0.035), had elevated NT-proBNP levels (*p* = 0.002) and higher need for catecholamines (*p* = 0.001). RBR was related to reduced GLS (*p* = 0.008), GCS (*p* = 0.031), GRS (*p* = 0.012), LV twist (*p* = 0.008), peak apical rotation (*p* < 0.001), and LVEF (*p* = 0.001), elevated LV end-diastolic volume (*p* = 0.023) and LV end-systolic volume (*p* = 0.003), and lower right ventricular stroke volume (*p* = 0.023).

**Conclusions:**

MWF was common, but failed to predict heart failure. RBR was associated with clinical and biventricular functional signs of heart failure as well as the occurrence of adverse events. Our findings suggest that RBR may predict outcomes and may serve as a novel marker of disease severity in pediatric DCM.

**Clinical Trial Registration:** https://clinicaltrials.gov/, identifier: NCT03572569.

## Introduction

Dilated cardiomyopathy (DCM) is the most common phenotype of cardiomyopathy in children and adolescents. Clinical courses are variable and range from asymptomatic to severe heart failure with significant morbidity and mortality especially in infants <1 year of age (1). Given the difficult clinical management and limited treatment options for advanced DCM, there is an urgent need for improved risk stratification to early identify patients at high risk for unfavorable outcomes.

Myocardial fibrosis has been appreciated as a key factor in the development of heart failure. Midwall fibrosis (MWF) as detected by cardiovascular magnetic resonance (CMR) late gadolinium enhancement (LGE) is present in ~25–35% of adult patients with non-ischemic DCM (2–5). Previous studies have shown that MWF is associated with ventricular dysfunction, arrhythmias, and cardiovascular death (2, 3). Moreover, failing response to pharmacologic treatment as well as to cardiac resynchronization therapy in DCM patients with MWF has been described (6, 7). While there is convincing evidence that MWF is a risk factor for adverse outcome in adult DCM, the prevalence and clinical relevance in pediatric patients remains unknown.

Strain, rotation and twist are important parameters of the cardiac performance. Deformation analyses with CMR feature tracking have shown that cardiac strain predicts survival and can refine risk stratification in adults with DCM (8). Opposite rotation of the apex and the base results in a twisting motion of the left ventricle (LV) essential for cardiac function (9). Impaired LV twist has been described as a marker of disease severity in adults with DCM (10). Recent reports have demonstrated the presence of rigid body rotation (RBR) with LV apex and base rotating in the same direction in adults with DCM and MWF (5) as well as in adult and pediatric LV non-compaction cardiomyopathy (11, 12). Data on cardiac mechanics particularly with regard to LV twist and RBR in children and adolescents with DCM are sparse to date (13).

The present study aimed to evaluate MWF and cardiac mechanics in pediatric primary DCM. We hypothesized that MWF and altered ventricular mechanics are predictive for heart failure in children and adolescents with DCM.

## Materials and Methods

### Study Population

We prospectively enrolled patients ≤21 years of age from the RIKADA study (NCT03572569) (14) with diagnosis of primary DCM (15) and clinically indicated CMR.

Exclusion criteria were myocardial inflammation (myocarditis), evidence of systemic disease with cardiac involvement (secondary CM), combination with other congenital heart diseases including congenital coronary artery anomalies, and contraindications to perform CMR.

### CMR Protocol

CMR was performed at 1.5 Tesla (Philips Healthcare, Best, The Netherlands). Cine images were acquired using a steady-state free precision sequence in long axis planes as well as in axial and short axis (SAX) orientation covering the entire ventricles. A T1-weighted inversion-recovery 3-dimensional spoiled gradient echo sequence was applied to obtain LGE images in identical long and SAX views 8 to 10 min after intravenous bolus injection of 0.2 mmol/kg of gadolinium-DOTA (Dotarem®, Guerbet). Inversion times were determined with an individually adapted prepulse delay Look-Locker sequence to null myocardium.

### Image Analysis

Volumetric parameters were measured using a commercially available workstation (Philips Viewforum, Best, the Netherlands). Image stacks in SAX were used for analysis of LV volumes, ejection fraction (EF), and mass, and in axial orientation for right ventricular (RV) functional data including end-diastolic volume (EDV), end-systolic volume (ESV), stroke volume (SV), and EF. Ventricular volumes and mass were indexed to body surface area (BSA).

#### LGE

The presence and pattern of LGE was determined blinded to clinical data. LGE was defined as an area with a signal intensity of >2 standard deviations (SD) above the mean intensity of normal myocardium in the same slice (2, 16). LGE characterized as MWF was only considered present if the area of signal enhancement was (a) confined to intramural and/or subepicardial layers, sparing the subendocardium, and (b) visible in both phase-encoding directions and in two orthogonal spatial orientations (2, 3). All participants were categorized according to the presence or absence of MWF (MWF+ vs. MWF–). LGE was quantified with a semi-automatic technique, using regions defined as above 50% of maximum signal intensity of the enhanced area (full-width at half maximum). LGE mass was then expressed as a percentage of total LV mass (17). LGE analysis was performed with QMass version 8.1 (Medis Medical Imaging Systems, Leiden, the Netherlands).

#### Strain

For feature tracking strain analysis, 2-, 3- and 4-chamber cine images as well as three preselected slices from the SAX image stacks corresponding to basal, midventricular and apical levels were considered. Using QStrain version 2.0 (Medis Medical Imaging Systems, Leiden, the Netherlands), endocardial and epicardial contours were drawn on the long and SAX cine images and detected throughout the whole cardiac cycle with a tissue tracking algorithm to calculate LV subendocardial global longitudinal strain (GLS), global circumferential strain (GCS), and global radial strain (GRS).

#### LV Rotation and Twist

Basal and apical SAX cine images were used to measure peak systolic rotation. LV twist was calculated as the difference between the peak rotation of the apex and the base at the same time point corresponding to the ejection phase in systole (11, 18). Counterclockwise rotation was assigned a positive value and clockwise rotation a negative value (11). Counterclockwise rotation at the apex and clockwise rotation at the base are typically seen in health (5). As previously described, systolic torsion was classified as: (a) normal torsion: predominantly anticlockwise rotation of the apex and clockwise rotation of the base; (b) RBR: both the apex and base rotating in the same direction; and (c) reverse torsion: predominantly clockwise rotation of the apex and anti-clockwise rotation of the base (5). Rotation analyses were performed using QStrain version 2.0 (Medis Medical Imaging Systems, Leiden, the Netherlands).

### Follow-Up

Patients were followed-up by medical record review. Mechanical circulatory support (MCS), heart transplantation (HTx) or listing for HTx, sudden cardiac death and all-cause death since the date of CMR were defined as adverse events.

### Statistical Analysis

Continuous values are given as median (range) or mean ± SD, as appropriate. Categorical data are presented as numbers (n) and percentages (%). Comparisons between groups were analyzed using the Mann-Whitney-U test for continuous variables and the chi square test or Fisher's exact test for categorical variables. For correlation analysis, the Spearman rank correlation coefficient was used. Results were considered significant at a *p*-value < 0.05. Analyses were performed with SPSS version 24.0 (IBM Corp., Armonk, NY).

## Results

**Figure 1:**
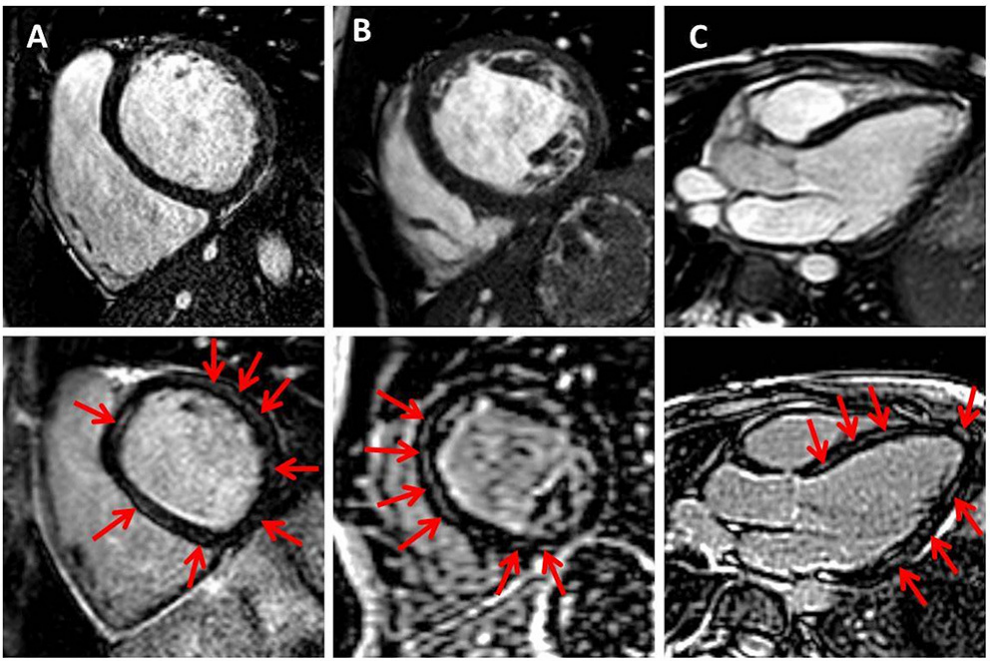
Midwall fibrosis in pediatric dilated cardiomyopathy. Exemplary cine (upper row) and corresponding late gadolinium enhancement (LGE) images (lower row) in basal and midventricular short axis **(A,B)** and in three-chamber view **(C)**. Red arrows indicate positive LGE.

Patient characteristics and CMR findings according to the presence of MWF are summarized in Tables 1, 2, respectively. In total, 17 DCM patients with a median age of 11.2 years at CMR were enrolled. In 7/17 patients (41%), MWF was observed (Figure 1). No patient showed areas of subendocardial or transmural LGE. Median N-terminal pro brain natriuretic peptide (NT-proBNP) levels were higher and LVEF lower in MWF+ than in MWF-, albeit without statistical significance (NT-proBNP: 5,959 vs. 242 pg/ml, *p* = 0.887; LVEF: 28 vs. 39%, *p* = 0.536). There were no significant differences between MWF+ and MWF– patients in age, gender, BSA, New York Heart Association (NYHA) classification, medication, and frequency of arrhythmias. The LGE percentage of LV mass was not associated with clinical data, ventricular function, deformation measures or LV twist.

**Table 1:** Patient characteristics.

	**Patients total (*n* = 17)**	**DCM MWF– (*n* = 10)**	**DCM MWF+ (*n* = 7)**	***P*-value**
Gender (female/male)	8 (47)/9 (53)	4 (40)/6 (60)	4 (57)/3 (43)	0.419
Age at CMR (years)	11.2 (6.1–13.9)	10.9 (7.9–15.4)	11.6 (3.4–12.8)	0.536
Age at diagnosis (years)	6.7 (0.2–13.0)	8.4 (0.2–13.9)	6.2 (0.3–12.8)	0.813
BSA (kg/m^2^)	1.3 (0.8–1.6)	1.2 (0.9–1.6)	1.3 (0.6–1.6)	0.813
NYHA
I II III IV n/a	8 (47) 0 (0) 4 (23.5) 1 (6) 4 (23.5)	6 (60) 0 (0) 2 (20) 0 (0) 2 (20)	2 (29) 0 (0) 2 (29) 1 (14) 2 (29)	0.466
VO_2_max (ml/min/kg)	28 (23–40) *n* = 6	23 (23–36) *n* = 5	45 *n* = 1	n.a.
NT-proBNP (pg/ml)	1,002 (48–9,475)	242 (51–12,746)	5,959 (10–9,520)	0.887
Medication^*^				
Heart failure	14 (82)	7 (70)	7 (100)	0.228
Catecholamines	8 (47)	4 (40)	4 (57)	0.637
Antiarrhythmic	2 (12)	0 (0)	2 (29)	0.154
Arrhythmias				
SVT	1 (93)	0 (0)	1 (17)	0.429
nsVT	3 (24) *n* = 14	1 (13) *n* = 8	2 (33) *n* = 6	0.538
MCS	4 (33)	2 (20)	2 (29)	1.000
HTx / listed for HTx	6 (35)/2 (12)	3 (30)/1 (10)	3 (43)/1 (14)	0.637
Death	1 (6)	1 (10)	0 (0)	1.000
Genetic variants				0.627
Pathogenic	0 (0)	0 (0)	0 (0)	
Likely pathogenic	2 (14)	1 (12.5)	1 (17)	
Unknown significance	7 (50)	3 (37.5)	4 (67)	
None	5 (36)	4 (50)	1 (17)	
	(*n* = 14)	(*n* = 8)	(*n* = 6)	

*Values are median (range) or n (%). Arrhythmias were documented with Holter-ECG*.

* *Medication at study enrollment*.

*Genetic variants were classified according to the guidelines of the American College of Medical Genetics and Genomics (19). Likely pathogenic variants were detected in sarcomere genes encoding for troponin T2 cardiac type (TNNT2) and titin (TTN), respectively (20)*.

*BSA, body surface area; CMR, cardiovascular magnetic resonance; DCM, dilated cardiomyopathy; HTx, heart transplantation; MCS, mechanical circulatory support; MWF–, no midwall fibrosis; MWF+, midwall fibrosis present; n/a, not available; nsVT, non-sustained ventricular tachycardia; NT-proBNP, N-terminal pro brain natriuretic peptide; NYHA, New York Heart Association; SVT, supraventricular tachycardia; VO_2_max, maximum oxygen consumption*.

**Table 2:** CMR findings according to the presence of midwall fibrosis.

	**Patients total**	**DCM MWF-**	**DCM MWF+**	***P*-value**
	**(*n* = 17)**	**(*n* = 10)**	**(*n* = 7)**	
LVEDV (ml/m^2^)	119 (96–211)	122 (108–207)	116 (89–224)	0.813
LVESV (ml/m^2^)	71 (46–177)	72 (47–168)	71 (44–199)	0.887
LVSV (ml/m^2^)	43 (29–56)	42 (35–58)	43 (20–56)	0.475
LVEF (%)	31 (18–52)	39 (19–53)	28 (16–48)	0.536
RVEDV (ml/m^2^)	94 (73–120)	97 (82–120)	80 (67–125)	0.740
RVESV (ml/m^2^)	51 (33–57)	48 (35–55)	51 (13–115)	1.000
RVSV (ml/m^2^)	44 (30–54)	49 (33–59)	40 (20–54)	0.230
RVEF (%)	56 (35–58)	57 (46–57)	50 (25–63)	0.417
LV global longitudinal strain (%)	-9.8 (-16.7 − -5.9)	-12.9 (-17.0 − -5.7)	-8.6 (-13.0 − -5.7)	0.713
LV global circumferential strain (%)	-10.0 (-23.2 − -6.4)	-14.5 (-23.9 − -6.9)	-8.4 (-15.4 − -5.2)	0.492
LV global radial strain (%)	39.9 (25.7–64.9)	45.4 (25.1–64.0)	38.7 (25.8–73.6)	1.000
LV twist (°)	5.6 (3.1–9.0)	5.2 (2.6–7.7)	5.8 (2.6–14.0)	0.713
LV peak basal rotation (°)	-6.7 (-8.7 − -1.6)	-3.9 (-7.6 − -1.5)	-8.7 (-9.4 − -4.7)	0.181
LV peak apical rotation (°)	-0.8 (-3.4–3.5)	0.2 (-3.3–3.7)	-3.0 (-4.3–4.7)	0.635
Rigid body rotation, n (%)	9/16 (56)	5 (50)	4/6 (67)	0.696

*Values are median (range) or n (%)*.

*LV, left ventricular; LVEDV, left ventricular end-diastolic volume; LVEF, left ventricular ejection fraction; LVESV, left ventricular end-systolic volume; LVSV, left ventricular stroke volume; MWF–, no midwall fibrosis; MWF+, midwall fibrosis present; RVEDV, right ventricular end-diastolic volume; RVEF, right ventricular ejection fraction; RVESV, right ventricular end-systolic volume; RVSV, right ventricular stroke volume*.

### Strain Analysis

MWF+ patients had reduced global GLS (*p* = 0.713), GCS (*p* = 0.492) and GRS (*p* = 1.000) in comparison to MWF– patients; but these differences were not statistically significant (Table 2).

Reduction of GLS, GCS, and GRS was observed in patients who were administered catecholamines (*p* = 0.001, 0.007, and <0.001, respectively) and in whom adverse events occurred (*p* < 0.001, <0.001, and 0.002, respectively).

### Rotation and Twist Analysis

**Figure 2:**
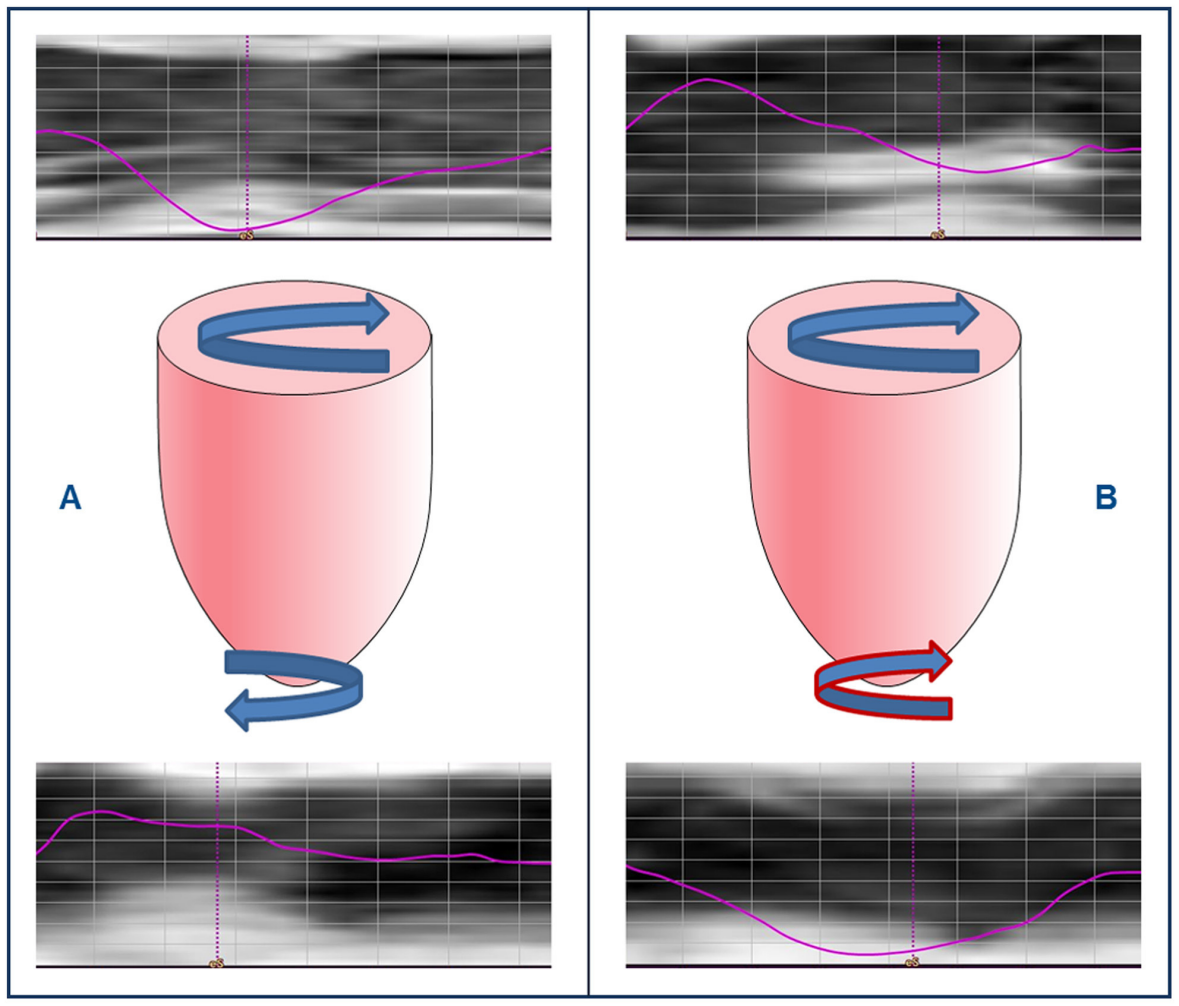
Rigid body rotation in pediatric dilated cardiomyopathy. Cardiovascular magnetic resonance feature tracking rotation curves of left ventricular (LV) base (upper row) and apex (lower row) in two pediatric patients with DCM, and schematic representations of LV rotation patterns (middle row). **(A)** LV twist with clockwise basal and counterclockwise apical rotation, and **(B)** rigid body rotation with base and apex rotating in the same direction.

Normal torsion was seen in 7/16 patients (44%), RBR in 9/16 patients (56%) (Figure 2), and reverse torsion in none. Rotation and twist could not be calculated in one patient due to incomplete datasets. Normal torsion was more frequently observed in MWF- than in MWF+ patients (50 vs. 33%), while RBR, resulting from reversed (clockwise) apical rotation was more common in MWF+ (67%). Patients with RBR more often were in the higher NYHA classes III and IV (80 vs. 14%, p = 0.035), had elevated NT-proBNP levels [9,429 (2,329–27,278) vs. 54 (25–337) pg/ml, *p* = 0.002], and higher need for catecholamines (89 vs. 0%, *p* = 0.001). Moreover, patients with RBR had reduced GLS (*p* = 0.008), GCS (*p* = 0.031), GRS (*p* = 0.012), LV twist (*p* = 0.008) and peak apical rotation (*p* < 0.001), reduced LVEF (*p* = 0.001), elevated LVEDV (*p* = 0.023) and LVESV (*p* = 0.003), and lower RVSV (*p* = 0.023) (Table 3). RBR was present in both patients with likely pathogenic variants in sarcomere genes encoding for troponin T2 cardiac type (TNNT2) and titin (TTN), respectively. The presence of RBR was not associated with age, gender, BSA, and frequency of arrhythmias.

**Table 3:** CMR findings according to the presence of rigid body rotation.

	**DCM RBR- (*n* = 7)**	**DCM RBR+ (*n* = 9)**	***P*-value**
LVEDV (ml/m^2^)	113 (80–119)	203 (128–239)	**0.023**
LVESV (ml/m^2^)	48 (44–69)	160 (102–205)	**0.003**
LVSV (ml/m^2^)	56 (37–65)	36 (25–44)	0.114
LVEF (%)	52 (33–56)	20 (13–25)	**0.001**
RVEDV (ml/m^2^)	101 (80–120)	87 (55–112)	0.299
RVESV (ml/m^2^)	45 (36–53)	51 (14–76)	0.758
RVSV (ml/m^2^)	51 (40–67)	32 (23–49)	**0.023**
RVEF (%)	56 (55–57)	49 (31–64)	0.918
LV global longitudinal strain (%)	-16.1 (-17.9 − -11.0)	-6.5 (-9.9 − -4.5)	**0.008**
LV global circumferential strain (%)	-22.9 (-26.0 − -10.5)	-7.7 (-10.7 − -5.3)	**0.031**
LV global radial strain (%)	55.2 (39.9–66.8)	25.8 (19.5–35.4)	**0.012**
LV twist (°)	9.7 (5.4–13.5)	4.0 (-1.0–5.8)	**0.008**
LV peak basal rotation (°)	-6.1 (-9.1 − -1.5)	-7.3 (-8.7 − -1.5)	0.837
LV peak apical rotation (°)	3.6 (1.0–4.7)	-3.3 (-4.5 − -2.2)	**<0.001**

*Values are median (range). Statistically significant p-values are indicated in bold*.

*LV, left ventricular; LVEDV, left ventricular end-diastolic volume; LVEF, left ventricular ejection fraction; LVESV, left ventricular end-systolic volume; LVSV, left ventricular stroke volume; RBR–, no rigid body rotation; RBR+, rigid body rotation present; RVEDV, right ventricular end-diastolic volume; RVEF, right ventricular ejection fraction; RVESV, right ventricular end-systolic volume; RVSV, right ventricular stroke volume*.

### Follow-Up

During a median follow-up time of 32 (12–46 months), 6/17 patients (35%) received HTx, with prior MCS in 4/6 patients; 2/17 patients were listed for HTx, and one patient died.

In the MWF- group, adverse events were seen in 4/10 patients (40%). Of those, three patients underwent HTx (prior MCS in 2/3 patients), and one patient was listed for HTx. One patient died due to hyperacute organ rejection 4.2 years post HTx. In the MWF+ group, adverse events occurred in 4/7 patients (57%). Of those, three patients underwent HTx (prior MCS in 2/3 patients), and one patient was listed for HTx.

Adverse events were more frequent in patients with RBR than in those with normal torsion (78 vs. 14%; *p* = 0.041). In total, 7/9 patients with RBR (78%) had adverse events during follow-up.

## Discussion

In this study, MWF appeared frequently in pediatric primary DCM (41%). We could see a tendency toward a more pronounced LV systolic dysfunction and reduction of strain as well as higher NT-proBNP levels in MWF+ patients. Our study for the first time assessed LV twist and RBR in pediatric DCM using CMR. RBR was more common in MWF+ patients, with a frequency of >50% in the overall patient cohort, and was related to clinical and biventricular functional markers of heart failure and to the occurrence of adverse events.

### Midwall Fibrosis

The findings of our study are comparable with previously published reports on adults with DCM in whom LGE was detected with a frequency of up to 39%, while others described lower presence of LGE (2–5). Data on LGE in children and adolescents with DCM are sparse (21, 22). In the study by Latus et al., LGE was present in only 16% of the pediatric DCM cohort despite marked LV dilation and dysfunction. LGE occurred in various patterns and was mostly detected in inflammatory DCM. The only patient with midwall LGE had idiopathic DCM (21), suggesting that MWF may be characteristic of non-inflammatory DCM not only in adults, but also in pediatric patients. Muscogiuri found LGE in 47% of children with DCM. In all patients, LGE occurred in a global diffuse subendocardial pattern compatible with primary endocardial fibroelastosis, and was associated with reduced global LV systolic function (22). In line with these data, it has been shown that LGE is related to functional impairment (23), and is a predictor of arrhythmias, cardiovascular hospitalization and death in adult DCM (2, 3). In our study, we could see a trend toward a more pronounced LV systolic dysfunction and reduction of GLS, GCS and GRS as well as higher NT-proBNP levels in MWF+ patients, but our results did not reach statistical significance. Moreover, RBR was more common in MWF+ patients, while normal torsion was more frequently observed in the MWF– group. This is in agreement with Taylor et al. who demonstrated RBR in adult patients with DCM and MWF. In that study, MWF was associated with reduced LV systolic and diastolic function, GCS, and twist, as well as the presence of RBR (5). We could see a tendency toward a higher probability of adverse events in MWF+ patients. However, the small sample size did not allow survival analysis. Our data indicate that MWF may be associated with clinical and functional signs of heart failure as well as altered cardiac mechanics in children and adolescents with DCM. Future research on larger cohorts is warranted to confirm our initial findings and analyze the potential prognostic implications of MWF in pediatric DCM. If the lack of statistical significance with regard to correlations between MWF and heart failure markers in our study is due to the small sample size or rather points to a minor clinical relevance of MWF in children and adolescents with DCM needs to be studied further.

### Cardiac Mechanics

Echocardiography studies have shown impaired LV rotation, torsion and twist in adults (10, 24, 25) and children with DCM (13), however, data on clinical implications of alterations in cardiac mechanics are sparse. Popescu et al. found reversed apical rotation and loss of LV torsion to be associated with significant LV remodeling, increased electrical dyssynchrony, reduced systolic function and increased filling pressures as indications for more advanced disease stages in adults with DCM (10). In ten children with DCM, the loss of LV torsion as measured by echocardiography was mainly due to the diminution of counterclockwise apical rotation and correlated with the degree of LV systolic dysfunction (13). Rotation of LV apex and base in the same direction (=RBR) has been demonstrated with echocardiography in adults (12, 26) as well as in children with LVNC (11).

To our knowledge, our study is the first assessing LV twist and RBR in pediatric DCM using CMR. We detected RBR, resulting from reversed clockwise apical rotation, in 56% of pediatric DCM patients. Our data are in agreement with the prevalence of 52% in adults described by Popescu et al. (10). We found RBR to be correlated with elevated NT-proBNP levels, and markers of LV dysfunction as well as impaired cardiac mechanics including reduced LV twist and apical rotation. Moreover, RBR was related to reduced RVSV, pointing to adverse ventriculo-ventricular interactions and the severity of disease in pediatric DCM patients. In line with higher NYHA functional classes in adults with LVNC and RBR (26), in our study children and adolescents with RBR were in higher NYHA classes than individuals with normal rotational patterns. We could demonstrate that the majority of patients with RBR experienced adverse events during follow-up (78%), suggesting that RBR may predict outcomes and may serve as an imaging marker of disease severity in pediatric DCM.

### Study Limitations

Our study has several limitations. Due to the small sample size, survival analysis could not be carried out. Although we could see a clear trend toward more pronounced LV dysfunction and higher NT-proBNP levels in MWF+ patients, a larger patient cohort may be needed to demonstrate statistically significant differences between MWF– and MWF+ patients with regard to clinical and functional markers of heart failure. Future prospective long-term studies are required to analyze the clinical importance of MWF in DCM potentially increasing with age. The study was conducted in a tertiary facility, offering all therapeutic options for end-stage heart failure, which may have led to an overrepresentation of severely ill DCM patients.

### Conclusions

In our study, we could demonstrate that MWF was a common feature in pediatric primary DCM, but failed to predict heart failure. The presence of RBR was related to clinical and biventricular functional markers of heart failure and to the occurrence of adverse events. Our findings suggest that RBR may predict outcomes and may serve as a novel marker of disease severity in pediatric DCM.

## Data Availability Statement

The raw data supporting the conclusions of this article will be made available by the authors, without undue reservation.

## Ethics Statement

The studies involving human participants were reviewed and approved by the Ethics Committee of the Charité - Universitätsmedizin Berlin. Written informed consent to participate in this study was provided by the participants' legal guardian/next of kin.

## Author Contributions

The authors contributed to the submitted work as follows: NA-W-M: conception and design of the study, acquisition, analysis and interpretation of data, and drafting the manuscript. FS: acquisition and analysis of data. JK: analysis and interpretation of data. TK and FB: conception and design of the study. SK and DM: conception and design of the study and interpretation of data. All authors contributed to manuscript revision, read, and approved the final manuscript.

## Funding

This work was supported by the DZHK (German Centre for Cardiovascular Research) (FKZ 81Z3100331 and FKZ 81Z0100301). We acknowledge support from the German Research Foundation (DFG) and the Open Access Publication Fund of Charité - Universitätsmedizin Berlin.

## Conflict of Interest

The authors declare that the research was conducted in the absence of any commercial or financial relationships that could be construed as a potential conflict of interest.

## Publisher's Note

All claims expressed in this article are solely those of the authors and do not necessarily represent those of their affiliated organizations, or those of the publisher, the editors and the reviewers. Any product that may be evaluated in this article, or claim that may be made by its manufacturer, is not guaranteed or endorsed by the publisher.
